# The poly (A) polymerase *pcnB* modulates virulence and resistance in *Klebsiella pneumoniae* by differentially regulating chromosomal mRNA stability and plasmid copy number

**DOI:** 10.3389/fmicb.2025.1709161

**Published:** 2025-12-11

**Authors:** Liaoqin Zhang, Shujie Zhang, Jun Wang, Zhongdong Zhang, Yong Xu, Chen Zhang

**Affiliations:** 1 School of Life Science, Anhui Agricultural University, Hefei, China; 2 Institute of Clinical Medicine, Anhui Academy of Medical Sciences, Hefei, China

**Keywords:** *Klebsiella pneumoniae*, pcnB, siderophore, EPS, antibiotic resistance, biofilm

## Abstract

*Klebsiella pneumoniae* is a leading cause of healthcare-associated infections, with emerging strains exhibiting both multidrug resistance and hypervirulence, largely mediated by plasmid-encoded genes. Poly (A) polymerase I, encoded by *pcnB*, plays a key role in RNA degradation and plasmid copy number control, yet its global regulatory impact in *K. pneumoniae* remains unclear. Here, we constructed a *pcnB* deletion mutant in *K. pneumoniae* ATCC 13883 using CRISPR-Cas9 and examined its effects on chromosomal virulence factors and plasmid-borne resistance. Deletion of *pcnB* impaired bacterial growth and metabolic activity, reduced biofilm formation, but unexpectedly enhanced siderophore and exopolysaccharide production via upregulation of chromosomal virulence genes. In contrast, *pcnB* deletion drastically reduced the copy number and stability of a ColE1-type plasmid carrying a spectinomycin resistance gene (*aadA*), leading to decreased *aadA* expression and a twofold reduction in antibiotic resistance. These findings reveal the dual role of *pcnB* as a repressor of chromosomal virulence genes and an activator of plasmid maintenance, highlighting its potential as a novel target for anti-virulence and anti-resistance strategies.

## 1 Introduction

*Klebsiella pneumoniae* (KP) is a Gram-negative opportunistic pathogen belonging to the Enterobacteriaceae family. As a common gut commensal, it ranks as the second most prevalent opportunistic pathogen in clinical settings (Assoni et al., 2021). This bacterium can cause various severe infections including pneumonia, meningitis, liver abscesses, and bloodstream infections (Breurec et al., 2016). In recent years, the widespread use of antibiotics has led to the emergence of KP strains exhibiting both multidrug resistance and hypervirulence (Holt et al., 2015). These KP strains result in significantly increased morbidity and mortality rates, and thereby impose considerable challenges on clinical treatment.

The pathogenicity of KP is attributed to multiple virulence factors, including capsular polysaccharides, siderophores, flagella, and resistance determinants such as carbapenemases (Chen et al., 2024). The expression levels of these factors directly influence the pathogenic capability of this bacterial species. Similar to *Escherichia coli* (*E. coli*), KP possesses a highly plastic genome capable of acquiring various resistance and virulence genes through horizontal gene transfer (Wyres et al., 2019). This characteristic facilitates the enhancement of virulence and development of antibiotic resistance, rendering conventional antibiotic therapies increasingly ineffective (Liu et al., 2021). Elucidating the transmission mechanisms of resistance and virulence genes, as well as deciphering the co-evolutionary relationship between bacteria and plasmids, will provide crucial theoretical basis for developing novel anti-bacterial strategies.

To address the dual challenges of multidrug resistance and hypervirulence in KP, significant efforts have been devoted to developing inhibitors targeting various pathogenic mechanisms, such as biofilm formation, drug efflux pumps, and *β*-lactamases (Lomovskaya et al., 2017; Mahrous et al., 2023; Vieira Da Cruz et al., 2023). Additionally, genetically disrupting key virulence mechanisms, including siderophore biosynthesis, capsule synthesis, and biofilm formation, has shown promising antibacterial effects (Han et al., 2022; Bina et al., 2023). However, current antimicrobial strategies often lack broad-spectrum efficacy, as most inhibitors target individual resistance or virulence genes, leaving other pathogenic mechanisms unaffected. In response to this limitation, ColE1-type plasmids have emerged as a new focus in the search for antibacterial targets, since these extrachromosomal elements are frequently identified as carriers of both resistance and virulence genes (Ares-Arroyo et al., 2021). Furthermore, experimental data have indicated that the presence of high-copy-number plasmids in KP is closely associated with multidrug resistance and hypervirulence phenotypes, playing a key role in its evolutionary trajectory (Ramirez et al., 2019). Therefore, targeting the mechanisms that regulate plasmid copy number could simultaneously disrupt the maintenance of all plasmid-borne resistance and virulence genes.

Based on current knowledge, the replication of ColE1-type plasmids is primarily regulated by the antisense RNA system involving RNA I and RNA II, while their copy number and stability are further modulated by poly (A) polymerase I (PAP I) (Cesareni et al., 1991). PAP I, encoded by the *pcnB* gene, was initially identified in *E. coli* for its ability to catalyze the addition of adenosine monophosphate (AMP) residues to the 3′ end of RNA, forming a poly (A) tail that decreases RNA stability and promotes its degradation (Lopilato et al., 1986; Jones, 2021). Subsequent studies revealed that PAP I plays a crucial role in controlling the copy number of ColE1-type plasmids in *E. coli* (Sarkar et al., 2002). Deletion of *pcnB* in *E. coli* leads to a sharp decrease in the copy number of endogenous ColE1-type plasmids (Wellner et al., 2025). Current evidence indicates that PAP I-mediated polyadenylation, encoded by *pcnB*, promotes the degradation of small RNAs that inhibit plasmid replication. Consequently, deletion of *pcnB* leads to the accumulation of these inhibitory sRNAs, resulting in decreased plasmid copy number and thereby reduced expression of plasmid-encoded genes (Schubert et al., 2025). However, the net impact of these opposing regulatory trends on plasmid gene expression remains unclear. Given that the high virulence and drug resistance of KP are primarily attributed to plasmid-encoded genes (Shankar et al., 2020), elucidating the regulatory role of *pcnB* in plasmid gene expression will provide important insights into the molecular mechanisms underlying KP virulence and drug resistance, and establish a theoretical basis for evaluating *pcnB* as a potential target for inhibiting bacterial virulence and resistance.

To address this, we selected the wild-type *K. pneumoniae* ATCC 13883 strain (Kp13883), which exhibits low virulence and high transformation efficiency, as our experimental model. By introducing an exogenous plasmid carrying a spectinomycin resistance gene and subsequently generating a *pcnB* knockout mutant using CRISPR-Cas9 technology, we systematically examined the differential regulatory effects of *pcnB* deletion on both chromosomally encoded virulence factors and plasmid-mediated drug resistance. This study aims to uncover the distinct regulatory mechanisms of *pcnB* on chromosomal and plasmid gene expression, thereby exploring its potential as a novel target for anti-infective drug development.

## 2 Materials and methods

### 2.1 Plasmids, bacterial strains, and growth conditions

The wild-type *Kp*13883 served as the parental strain in this study. All strains and plasmids used are listed in Table S1. The wild-type strain, its *pncB* deletion mutant, and the complementation strain were designated as WT, Δ*pcnB* and Δ*pcnB* (pBBR-*pcnB*), respectively. Strains harboring the ColEI-type plasmid pEcgRNA (spectinomycin resistant) were denoted as WT- pEcgRNA and Δ*pcnB*-pEcgRNA. Bacterial cultures were routinely grown in Luria-Bertani (LB) medium and M9 minimal medium (Table S2) at 37 °C with 180 rpm shaking or on LB agar plates overnight. For strains carrying temperature-sensitive plasmids, a 42 °C incubation was applied to facilitate plasmid curing. When required, antibiotics were supplemented at the following concentrations: 50 μg/mL kanamycin (Kan), spectinomycin (Spec), and apramycin (Apr).

### 2.2 Preparation of competent cells and transformation

Competent cells were prepared using an optimized arabinose induction protocol. Briefly, *Kp*13883 overnight culture was diluted 1:50 in fresh LB medium and incubated at 37 °C with shaking until reaching OD_600_ of 0.2–0.3. L-arabinose was then added to a final concentration of 0.1% (w/v) to induce competence-related genes. Upon reaching OD_600_ of 0.5, cells were harvested by centrifugation at 4,000 × g for 10 min at 4 °C and washed four times with ice-cold 10% glycerol. Finally, the cell pellet was resuspended in a minimal volume of 10% glycerol and either used immediately or stored at −80 °C (Jiang et al., 2016; McConville et al., 2021).

For electroporation, 1 μg plasmid DNA was mixed with 100 μL competent cells on ice. The mixture was transferred to a pre-chilled 2-mm electroporation cuvette and pulsed at 2.5 kV using a Gene Pulser system (Bio-Rad). Immediately after electroporation, 900 μL SOC recovery medium was added, and cells were incubated at 37 °C for 1 h with shaking. Aliquots (10 μL and 50 μL) were plated on LB agar containing appropriate antibiotics and incubated overnight at 37 °C.

### 2.3 Plasmid construction of mutagenesis

All PCR primers were designed using SnapGene software (v5.2) based on the *Kp*13883 genomic sequence (NCBI accession no. CP009208) and are listed in Table S1. The *pcnB* deletion mutant was generated using pCasKP-apr/pSGKP-km mediated CRISPR-Cas9 editing system as previously described (Sun et al., 2019), with modifications. Specifically: (i) Two ~ 600-bp homology arms flanking the *pcnB* gene were amplified as a single fragment and cloned into pSGKP-km. (ii) Guide RNA (gRNA) targeting *pcnB* was designed using CHOPCHOP online tool (ref) (Labun et al., 2019) and cloned into pSGKP-km plasmid. (iii) The constructed plasmids were sequentially transformed into *Kp*13883 harboring pSGKP-km through electroporation. (iv) Positive clones were selected on LB plates containing triple antibiotics (Kan and Apr) and verified by colony PCR using *pcnB*-specific primers (P1/P2 in Table S1) and sequencing.

### 2.4 Supplementation of pcnB mutants

To confirm that the observed phenotype was caused by the *pcnB* gene deletion. The coding sequence of the *pcnB* gene was first amplified fromWT strain genomic DNA using specific primers (Table S1). These primers were designed with 20-bp homologous arms at their 5′ ends complementary to the termini of the pBBR1MCS-2 vector linearized by inverse PCR. The purified *pcnB* PCR fragment was then inserted into the multiple cloning site (MCS) of the linearized vector, with *pcnB* gene expression driven by the vector’s native lac promoter and ribosome binding site (RBS). Assembly was performed using the Ultra-Universal One Step Seamless Cloning Mix kit (CWBIO, China) at 50 °C for 15 min. The assembled product was transformed into *E. coli* DH5α competent cells and plated on LB agar plates containing kanamycin for selection. Finally, positive clones were verified by colony PCR. The plasmid was then extracted and electroporated into the Δ*pcnB* strain to construct the complementing strain Δ*pcnB* (pBBR-*pcnB*), which was finally validated by bacterial suspension PCR.

### 2.5 Bacterial growth curve analysis

WT, Δ*pcnB* and Δ*pcnB* (pBBR-*pcnB*) strains were cultured overnight in LB medium at 37 °C with 180 rpm shaking. Three biological replicates per strain were diluted to an OD_600_ of 0.1 in fresh LB medium. Growth was monitored by measuring OD_600_ values at 1-h intervals for 12 h under identical culture conditions, followed by growth curve construction.

### 2.6 Biofilm formation assay and viability testing

Overnight cultures in LB medium were adjusted to 0.5 McFarland standard. Aliquots (10 μL) of bacterial suspension plus 190 μL fresh LB medium were added per well in 96-well plates (*n* = 6 biological replicates). After 36-h incubation at 37 °C, planktonic cells were removed and wells were washed twice with PBS. Biofilms were fixed with 200 μL 4% paraformaldehyde for 30 min, air-dried, then stained with 0.1% crystal violet (200 μL, 20 min). Excess stain was removed by PBS washing, and bound dye was solubilized with 200 μL 33% glacial acetic acid for spectrophotometric measurement at 570 nm (LB medium as negative control).

Bacterial viability was assessed using fluorescein diacetate (FDA) which serves as a substrate for detecting metabolically active bacteria. Its hydrolysis by intracellular esterases generates fluorescent products, allowing quantitative measurement of hydrolytic enzyme activity in bacterial biofilms and subsequent evaluation of metabolic vitality. A 10 mg/mL FDA stock solution (in organic solvent) was prepared for light-protected storage. Working solution was prepared fresh in PBS with vortex mixing. Cultures at logarithmic and stationary phases were OD_600_-normalized and tested in triplicate. Reaction mixtures (50 μL total) containing 45 μL FDA working solution and 5 μL bacterial suspension were incubated at 37 °C for 20 min before fluorescence spectrophotometry. The excitation and emission wavelengths for FDA detection are 490 nm and 520 nm, respectively.

### 2.7 Siderophore quantification

Siderophore production was assessed using the Chrome Azurol S (CAS) assay (Rathod et al., 2024). The CAS solution was prepared by combining 2 mM CAS, 1 mM FeCl_3_·6H_2_O (in 10 mM HCl), and piperazine buffer (pH 5.6) with Hexadecyltrimethylammonium bromide (HDTMA) in a 100-mL cylinder. A 0.2 M sulfosalicylic acid shuttle solution was used as the iron chelator. Bacterial cultures were grown to log or stationary phase in low-phosphate minimal medium buffered with HEPES (to avoid phosphate interference). For detection, 0.5 mL CAS solution was mixed with 0.5 mL culture supernatant, followed by 10 μL shuttle solution. After 5 min incubation, absorbance at 630 nm was measured against appropriate controls. Siderophore units were calculated as [(A_n_-A_s_)/A_n_] × 100, where A_n_ and Aₛ represent reference and sample absorbances, respectively.

### 2.8 Extracellular polysaccharide (EPS) measurement

EPS was quantified by anthrone-sulfuric acid method (Jing et al., 2022). Overnight cultures (7 mL) were centrifuged (5,000 × g, 15 min), washed with PBS, then treated with 0.1 M NaOH (2 h, room temperature). After centrifugation (3,500 × g, 15 min, 4 °C), supernatants were mixed with 0.2% anthrone-sulfuric reagent (1:3 ratio; 0.2 g anthrone in 100 mL concentrated H_2_SO_4_), heated at 100 °C for 7 min, and measured at 630 nm after cooling.

### 2.9 Plasmid copy number determination

Log-phase bacterial cultures were harvested after OD_600_ normalization, and total DNA was extracted for relative quantification. Plasmid copy numbers (PCN) were calculated by qPCR using a chromosome-encoded reference gene *gapA* for normalization (Hao et al., 2025). To specifically target the plasmid, we designed a pair of qPCR primers (Table S1) amplifying a 150-bp fragment within the SpecR (*aadA* gene encoding an aminoglycoside adenyltransferase) gene of pEcgRNA. and relative copy number was calculated using the comparative threshold cycle 2^−ΔΔCt^ method.

### 2.10 Minimum inhibitory concentration (MIC) testing

The optimized broth microdilution method was used to determine the MICs of Spec against the WT, Δ*pcnB*, WT-pEcgRNA, and Δ*pcnB*-pEcgRNA strains. The assay adhered to the fundamental framework of the Clinical and Laboratory Standards Institute (CLSI) guidelines, with a refinement step incorporated to enhance accuracy. Initially, a standard two-fold serial dilution was performed for preliminary screening to determine the approximate MIC range (e.g., testing concentrations of 256, 128, 64, 32, and 16 μg/mL). Subsequently, a more concentrated gradient dilution was conducted within the critically identified range to precisely determine the MIC value. During the experiment, within the range of 64 μg/mL to 16 μg/mL, a series of intermediate concentrations (e.g., 56, 48, 40, 32, and 24 μg/mL) were additionally tested. Three technical replicates and blank controls were included per condition. The MIC was defined as the lowest antibiotic concentration preventing visible growth after incubation.

### 2.11 RNA quantification and gene expression analysis

Total RNA was extracted using Bacterial RNA Kit (Tiangen, China) and reverse-transcribed with FastKing RT Kit (Tiangen, China) following manufacturer’s protocols. Quantitative PCR was performed on an ABI 7500 Real-Time PCR System (Applied Biosystems, USA) using 16S rRNA as endogenous control. Relative gene expression was detected using specific primers (Table S1) and calculated via 2^−ΔΔCt^ method.

## 3 Results

### 3.1 Construction of ΔpcnB mutant, off-target detection and growth analysis

The *pcnB* gene in *Kp*13883 was precisely deleted using the CRISPR-Cas9 editing system. Colony PCR screening followed by sequencing confirmed the successful generation of mutant strain (Δ*pcnB*) with complete open reading frame deletion. In addition, we also constructed the *pcnB*-complemented strain (Figure S1).

To validate the specificity of the target sequence, potential off-target sites were identified using CAS-OFFinder (allowing up to 4 mismatches). PCR amplification was then performed using genomic DNA from the Δ*pcnB* strain. The successful amplification of correctly sized products (~1,200 bp) from all predicted off-target sites indicates the absence of substantial off-target editing at these loci (Figure S1; Table S3).

**Figure 1:**
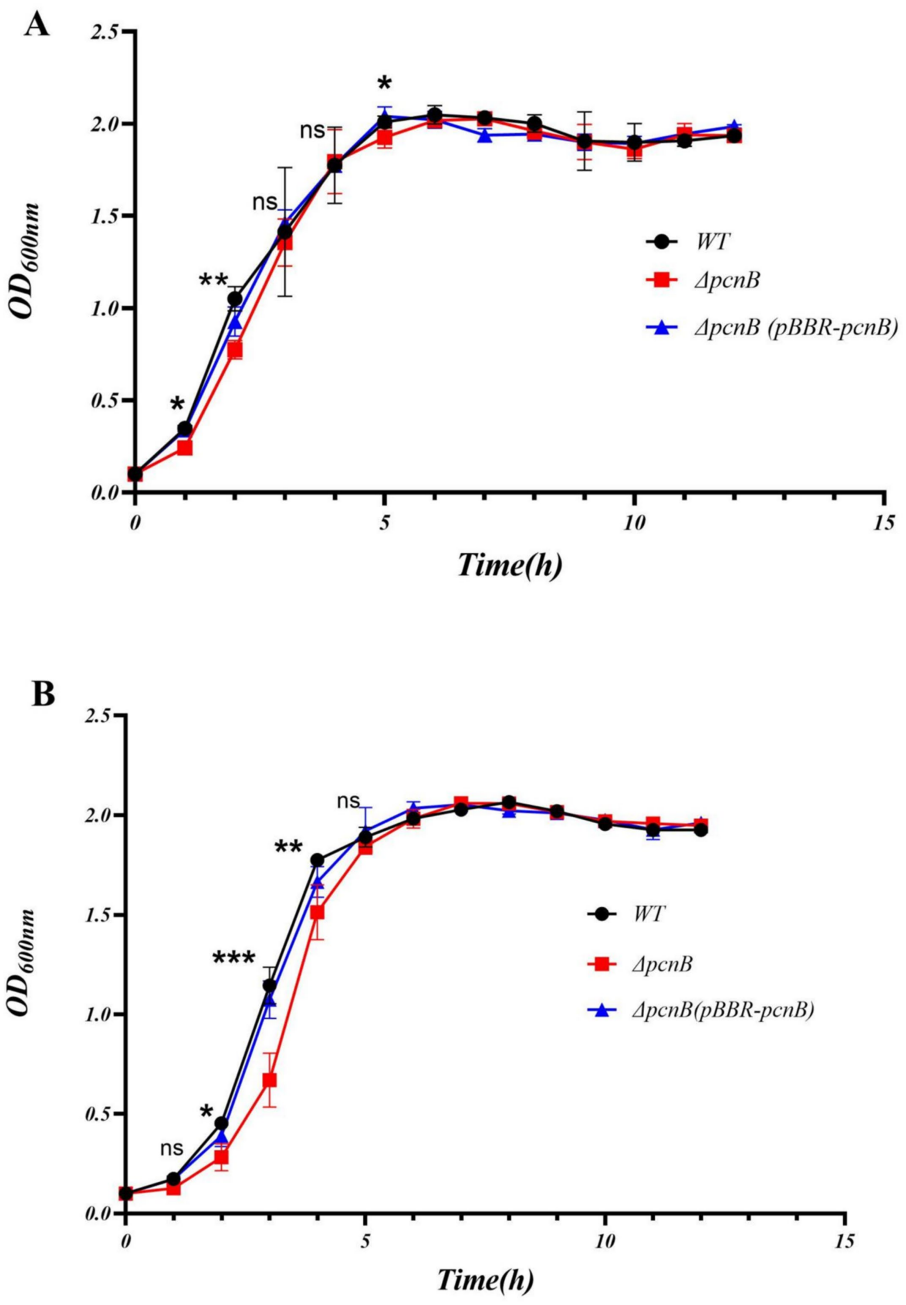
Growth curves of the WT, Δ*pcnB* and Δ*pcnB* (pBBR*-pcnB*) strains in LB **(A)** and M9 medium **(B)**. The black curve represents the WT strain, the red curve represents the Δ*pcnB* strain, and the blue curve represents the complement strain Δ*pcnB* (pBBR-*pcnB*). The bacteria were shaken at 37 °C (180 rpm), and the OD_600_ value was measured every hour to draw the growth curve. The WT and Δ*pcnB* strains were compared using a two-tailed Student’s *t*-test at the 1, 2, 3, 4, and 5 h time points. The error bars represent the SEM (*n* = 3, **, *p* < 0.01 relative to WT).

To investigate the physiological impact of *pcnB* deletion, we compared growth kinetics WT, Δ*pcnB* and Δ*pcnB* (pBBR-*pcnB*) strains in nutrient-rich LB and minimal M9 media. Starting from standardized inocula (initial OD_600_ ≈ 0.1), culture densities were monitored spectrophotometrically at 1-h intervals for 12 h. Growth curve analysis revealed that in LB medium, although the Δ*pcnB* mutant entered log-phase normally, its maximum specific growth rate (μ_max) was 13.6% lower than WT (*p* < 0.01), while theΔ*pcnB* (pBBR-*pcnB*) strain showed a significant but smaller reduction of 7.8% compared to the Δ*pcnB* (*p* < 0.05) (Figure 1A). Notably, three strains reached stationary phase after 4 h with comparable final OD_600_ absorption (*p* > 0.05) indicating *pcnB* deletion does not affect ultimate biomass accumulation.

More pronounced growth defects were observed in M9 minimal medium (Figure 1B). Compared to the WT, the Δ*pcnB* strain exhibited a 22.3% reduction in μ_max (*p* < 0.001) with prolonged logarithmic phase duration, and the Δ*pcnB* (pBBR-*pcnB*) strain exhibited a 5.7% reduction. This growth retardation suggests impaired metabolic efficiency under nutrient-limiting conditions, but the Δ*pcnB* (pBBR-*pcnB*) was able to partially restore this impairment. However, the growth defect of the Δ*pcnB* strain was compensated upon entering stationary phase, with no significant differences in growth parameters observed at other time points, compared with the WT strain.

### 3.2 Modulation of pcnB in cellular viability and biofilm formation in KP

**Figure 2:**
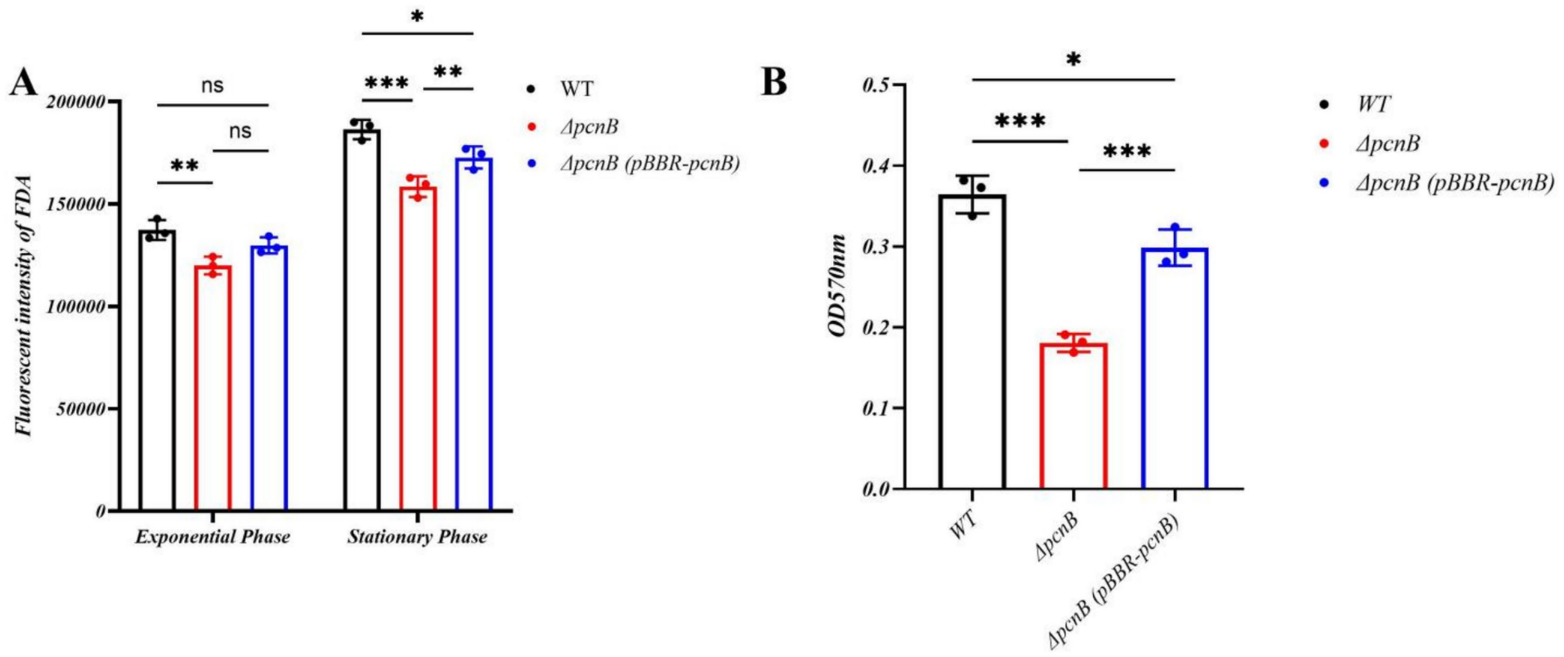
Bacterial vitality and biofilm formation ability of WT, Δ*pcnB* and Δ*pcnB* (pBBR-*pcnB*) strains. **(A)** Comparison of bacterial vitality in WT, Δ*pcnB* and Δ*pcnB* (pBBR-*pcnB*) strains at both the logarithmic and stationary growth phases. **(B)** Biofilm formation ability of WT, Δ*pcnB* and Δ*pcnB* (pBBR-*pcnB*) strains. Data points represent the mean of three biological replicates, each of which was averaged from three technical replicates. The error bars represent the SEM. Statistics: two-way ANOVA **(A)** and one-way ANOVA **(B)**. ns, *p >* 0.05; *, *p* < 0.05; **, *p* < 0.01; ***, *p* < 0.001.

To further elucidate the physiological role of *pcnB*, we assessed metabolic activity using fluorescein diacetate (FDA) hydrolysis assays. Synchronized measurements of OD_600_ and FDA fluorescence (excitation/emission: 490/520 nm) were carried out. To account for differences in cell density, FDA fluorescence intensities were normalized to the corresponding OD_600_ values. As shown in Figure 2A, the WT strain exhibited significantly higher metabolic activity than Δ*pcnB* in LB medium, and importantly, the Δ*pcnB* (pBBR-*pcnB*) strain partially restored this activity in the Δ*pcnB*. During the logarithmic phase, the fluorescence intensity of WT (137,330 ± 4,856 a.u.) was 12.6% higher than that of Δ*pcnB* (119,944 ± 4,274 a.u.; *p* < 0.01), and 5.4% higher than that of the Δ*pcnB* (pBBR-*pcnB*) strain (129,809 ± 3,976 a.u.; *p* > 0.05). This disparity further increased in the stationary phase, where WT reached 186,392 ± 4,765 a.u. compared to 158,509 ± 5,034 a.u. for Δ*pcnB* (*p* < 0.001), representing a 15% reduction in metabolic activity upon *pcnB* deletion, while Δ*pcnB* (pBBR-*pcnB*) strain (172,742 ± 5,342 a.u; *p* < 0.05) partially restored the activity, demonstrating a gene dosage effect.

Given that biofilm formation is directly influenced by bacterial viability and plays a crucial role in antibiotic resistance, we next investigated whether the observed reduction in cell viability affected biofilm development. To investigate the effect of *pcnB* on biofilm formation in KP, we quantified the biofilm biomass of WT, Δ*pcnB* and Δ*pcnB* (pBBR-*pcnB*) strains using crystal violet staining. OD_570_ measurements revealed that after 36 h of cultivation in LB medium, the biofilm OD_570_ value of WT was 0.364 ± 0.02, In contrast, the value forΔ*pcnB* (pBBR-*pcnB*) was reduced to 0.298 ± 0.02 (*p* < 0.05), while that of Δ*pcnB* was significantly reduced to 0.181 ± 0.01 (p < 0.001) (Figure 2B). Although three strains exhibited relatively weak biofilm-forming capacity in LB medium (OD_570_ < 0.4), *pcnB* deletion still resulted in a 50% reduction in biofilm formation, demonstrating that *pcnB* positively regulates biofilm development in KP.

### 3.3 Effect of pcnB deletion on chromosomal virulence gene expression in KP

To elucidate the regulatory role of *pcnB* deletion in chromosomally encoded virulence factors of KP, we quantified the production of siderophores and EPS in WT, Δ*pcnB* (pBBR-*pcnB*) and Δ*pcnB* strains.

**Figure 3:**
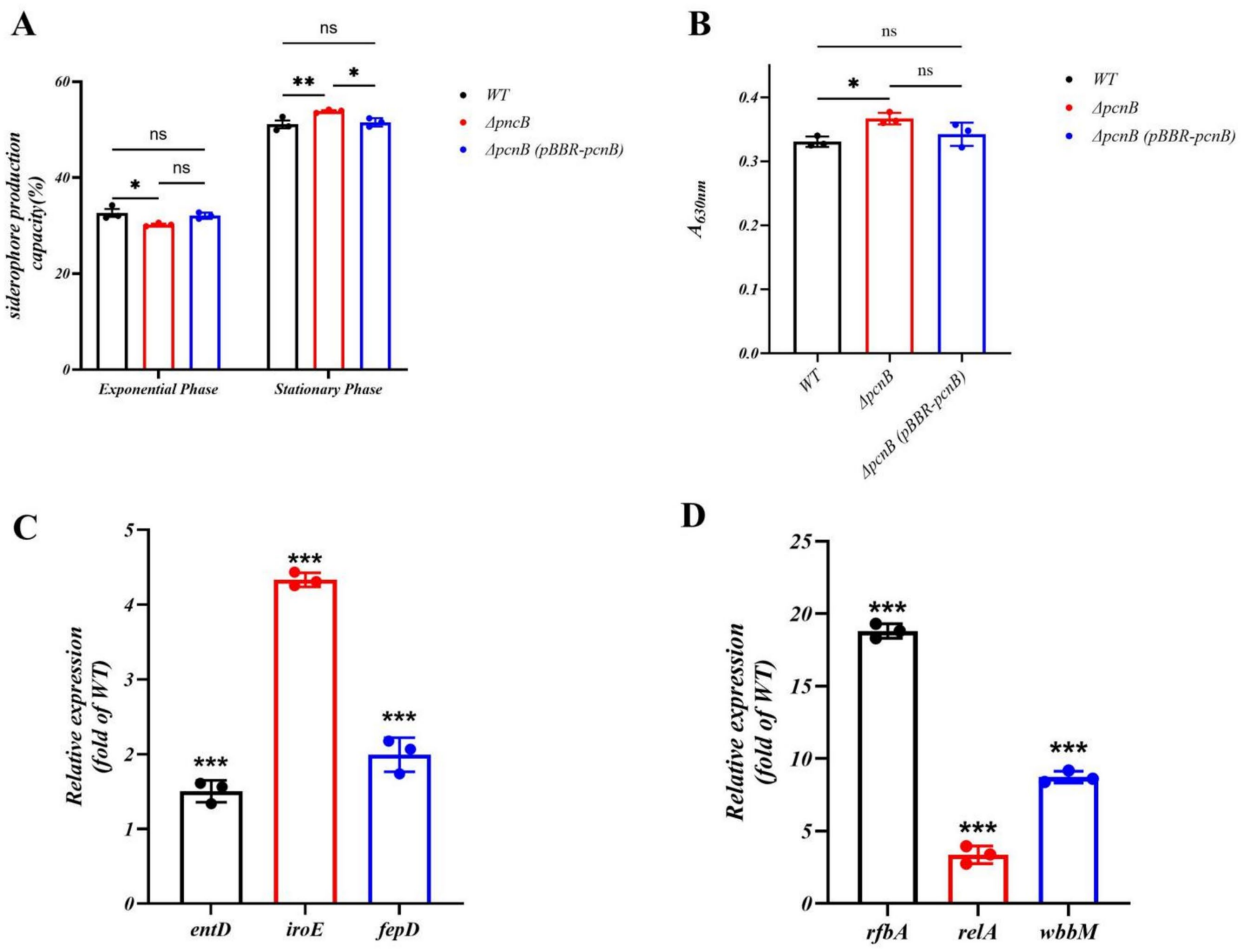
Changes in bacterial virulence factor and related gene expression. **(A)** Comparison of bacterial siderophore content in WT, Δ*pcnB,* and Δ*pcnB* (pBBR-*pcnB*) strains at both the logarithmic and stationary growth phases. **(B)** Comparison of bacterial EPS content in WT, Δ*pcnB,* and Δ*pcnB* (pBBR-*pcnB*) strains. **(C)** Relative mRNA levels of *entD*, *fepD*, and *iroE* in the Δ*pcnB* strain versus WT strain. **(D)** Relative mRNA levels of *rfbA*, *relA,* and *wbbM* in Δ*pcnB* strain versus WT. Data points represent the mean of three biological replicates, each of which was averaged from three technical replicates. The error bars represent the SEM. Statistics: two-way ANOVA **(A)**, one-way ANOVA **(B)** and *t*-test **(C,D)**. ns, *p* > 0.05; *, *p* < 0.05; **, p < 0.01; ***, p < 0.001.

The chrome azurol S (CAS) assay revealed that during logarithmic growth, the iron-chelating capacity of Δ*pcnB* showed significant difference compared to WT (*p* < 0.05), while the Δ*pcnB* (pBBR-*pcnB*) strain showed a partial restoration (*p* > 0.05; Figure 3A; Figure S3). However, upon entering stationary phase, Δ*pcnB* exhibited a 5.4% increase (*p* < 0.01) in siderophore units relative to WT. Moreover the siderophore level in Δ*pcnB* was also 3.7% higher than that of the Δ*pcnB* (pBBR-*pcnB*) strain (p < 0.05). indicating enhanced iron acquisition capability upon *pcnB* deletion during late growth phase. Anthrone-sulfuric acid assay for EPS quantification demonstrated that after OD_600_ normalization, the insoluble EPS yield in Δ*pcnB* (0.367 ± 0.01) was 0.1-fold higher than WT (0.331 ± 0.01). EPS production in the Δ*pcnB* (pBBR-*pcnB*) strain was somewhat lower than that in the Δ*pcnB* strain (0.342 ± 0.01; Figure 3B).

Quantitative PCR analysis showed upregulation of siderophore biosynthesis genes *entD*, *fepD*, and *iroE* in Δ*pcnB* by 1.5-fold, 1.8-fold, and 4-fold, respectively, (Figure 3C). More pronounced upregulation was observed in EPS synthesis genes *rfbA*, *relA*, and *wbbM*, with 18-fold, 3-fold, and 8-fold increases, respectively, (Figure 3D). These findings demonstrate that *pcnB* deletion enhances KP’s siderophore and EPS production through transcriptional activation of virulence-associated genes.

### 3.4 Effect of pcnB deletion on spectinomycin resistance of KP mediated by pEcgRNA

Considering the potential for intrinsic spectinomycin resistance on the KP chromosome, we sought to rule out any genome background-associated increase in basal resistance post-knockout. We therefore measured the spectinomycin MIC for WT and Δ*pcnB*. Table 1 shows that without pEcgRNA, the MIC of WT was 48 μg/mL, significantly greater than the 32 μg/mL of Δ*pcnB*, suggesting that *pcnB* deficiency also compromises KP’s intrinsic antibiotic tolerance.

**Table 1:** MICs for WT and Δ*pcnB.*

KP strain	Antibiotic	MIC (in μg/mL)
WT	Spec	48 μg/mL
Δ*pcnB*	Spec	32 μg/mL

To investigate the role of *pcnB* in regulating exogenous resistance plasmids, we introduced the ColE1-type plasmid pEcgRNA (carrying the *aadA* gene encoding an aminoglycoside adenylyltransferase) into WT and Δ*pcnB* strains (designated WT-pEcgRNA and Δ*pcnB*-pEcgRNA). Broth microdilution assays revealed that the Spec MIC of WT-pEcgRNA was 11 mg/mL, whereas Δ*pcnB*-pEcgRNA exhibited a 2-fold reduction in MIC (Table 2), In summary, *pcnB* knockout concurrently reduced both chromosomal basal tolerance and plasmid-mediated acquired resistance. However, the difference between the respective decreases (12 μg/mL vs. 11 mg/mL) is negligible, indicating compromised spc resistance upon *pcnB* deletion.

**Table 2:** MICs for WT-pEcgRNA and Δ*pcnB*-pEcgRNA.

KP strain	Antibiotic	MIC (in mg/mL)
WT- pEcgRNA	Spec	22 mg/mL
Δ*pcnB*-pEcgRNA	Spec	11 mg/mL

**Figure 4:**
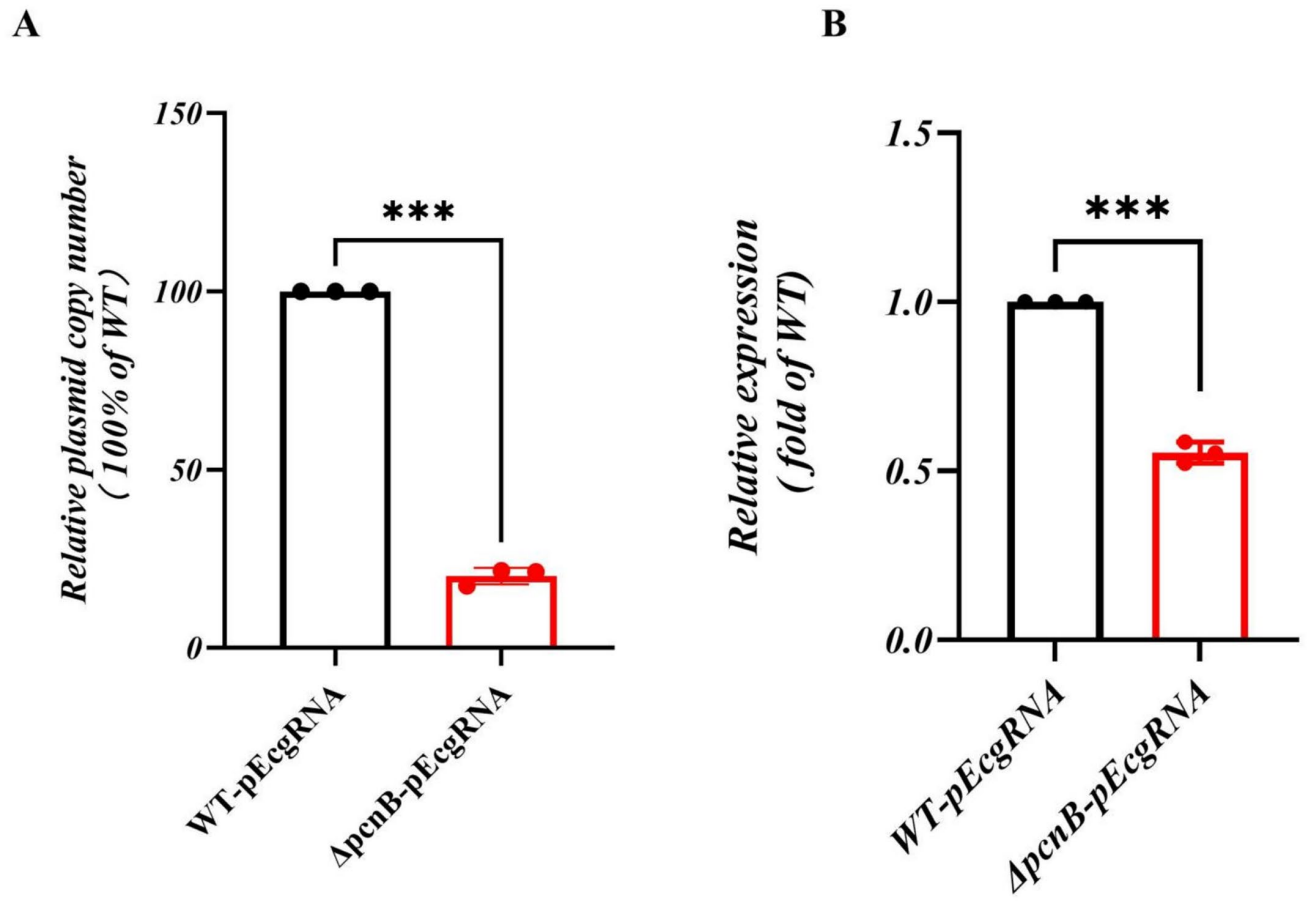
Relative plasmid copy number and expression of plasmid-encoded genes in WT versus Δ*pcnB* strains. **(A)** Relative plasmid copy number of pECgRNA in Δ*pcnB* strain versus WT strain. The copy number was determined by qPCR using the chromosomal *gapA* gene for normalization and is presented relative to the WT strain, which was set to 100%. **(B)** Relative gene expression of *aadA* in Δ*pcnB* strain versus WT strain. Data points represent the mean of three biological replicates, each of which was averaged from three technical replicates. The error bars represent the SEM. Statistics: *t*-test. ns, *p* > 0.05; *, *p* < 0.05; **, *p* < 0.01; ***, *p* < 0.001.

Quantitative PCR (qPCR) analysis of plasmid copy number (PCN), using the single-copy chromosomal gene *gapA* for normalization, demonstrated an 80% decrease in PCN for Δ*pcnB*-pEcgRNA compared to WT-pEcgRNA (Figure 4A). Additionally, transcriptional analysis of the *aadA* gene (normalized to 16S rRNA) showed a 45% reduction in expression in Δ*pcnB*-pEcgRNA (Figure 4B). These results suggest that *pcnB* deletion diminishes Spec resistance by reducing both ColE1 plasmid stability and resistance gene expression.

## 4 Discussion

In recent years, the increasing prevalence of hypervirulent and multidrug-resistant KP, particularly the emergence of carbapenem-resistant hypervirulent KP (CR-hvKP), has posed significant challenges to clinical anti-infective therapy (Turton et al., 2019). The pathogenicity of these strains is commonly mediated by plasmid-carried virulence and resistance genes, making the study of key host factors regulating plasmid stability and gene expression crucial for developing novel antibacterial strategies. This study focused on the *pcnB* gene, which encodes PAP I that participates in both RNA degradation regulation and direct control of ColE1-type plasmid copy number. Using CRISPR-Cas9 technology, we successfully constructed a *pcnB* deletion mutant and systematically evaluated its dual functions in regulating chromosomal virulence factor expression and exogenous plasmid-mediated resistance. Moreover, the subsequent construction of Δ*pcnB* (pBBR-*pcnB*) strain, which partially restored the key phenotypic defects, strongly supports the conclusion that *pcnB* plays a central role in coordinating these physiological processes.

Building on the previously reported role of *pcnB* (PAP I) in mRNA degradation, our results demonstrated (Figures 1A,B) that *pcnB* deletion significantly affected bacterial physiological states. In terms of growth characteristics, the Δ*pcnB* strain showed markedly reduced maximum growth rate during the logarithmic phase, with more pronounced delay in minimal M9 medium. This phenomenon suggests that *pcnB* deletion may impair metabolic efficiency. Notably, although logarithmic growth retardation, the stationary phase biomass remained unchanged, it exhibits a growth lag rather than a permanent growth defect, which is consistent with the observation by Francis & Laishram that the Δ*pcnB* strain displays a dynamic adaptive capacity due to increased mRNA stability under nutrient-restricted conditions (Francis and Laishram, 2021). Furthermore, normal metabolic activity may be sustained through compensatory mechanisms mediated by other RNA-metabolizing enzymes (such as RNase R or PNPase) (Gerdes et al., 2003; Babina et al., 2023). This growth delay likely results from gene expression reprogramming caused by altered RNA stability.

Regarding virulence and plasmid-mediated stress tolerance regulation, *pcnB* deletion exhibited a remarkable “double-edged sword” effect. On one hand, siderophore and EPS production significantly increased during stationary phase (Figures 3A,B), accompanied by elevated transcription levels of related genes (e.g., *entD*, *fepD*, *iroE*, *rfbA*, *relA*, *wbbM*) (Figures 3C,D). These data suggest that *pcnB* may indirectly suppress virulence factor expression by promoting degradation of these virulence-associated mRNAs. By promoting the polyadenylation of structured RNAs, *pcnB* ensures their efficient turnover; its absence leads to the accumulation of these transcripts, resulting in genome-wide expression dysregulation and pleiotropic effects on bacterial physiology (Maes et al., 2017). These results provide the first molecular evidence that *pcnB* serves as a repressive regulator in the KP chromosomal virulence network. On the other hand, *pcnB* deletion caused an 80% reduction in ColE1-type plasmid copy number (Figure 4A), 45% decrease in *aadA* resistance gene expression (Figure 4B), and a corresponding 2-fold reduction in the spectinomycin MIC (Table 2). These results clearly demonstrate the strong positive regulatory role of *pcnB* in plasmid maintenance and resistance transmission. Thus, *pcnB* is essential for the high-level antibiotic resistance mediated by ColE1-type plasmids. This finding is highly consistent with recent studies in *Escherichia coli*, further supporting the conserved function of *pcnB* in plasmid biology among Enterobacteriaceae (Schubert et al., 2025; Wellner et al., 2025).

Particularly noteworthy is that the dual mechanisms of *pcnB* in regulating both plasmid copy number and mRNA stability explain its “contradictory” roles in gene expression regulation (Schubert et al., 2025). For specific chromosome-encoded genes, *pcnB* deletion increases their mRNA stability and expression levels by impairing polyadenylation-mediated RNA decay. Conversely, for plasmid-encoded genes, *pcnB* deletion decreases plasmid copy number by stabilizing antisense RNAs that inhibit replication, thereby indirectly reducing gene dosage. Although reduced plasmid copy number should theoretically decrease gene dosage, the concomitant loss of mRNA degradation function in Δ*pcnB* actually increases transcript stability for some plasmid genes (e.g., *aadA*), resulting in phenotypic changes smaller than copy number variations. This complex regulatory pattern suggests that *pcnB* occupies a central hub position in bacterial gene expression networks, fine-tuning gene expression levels by coordinating gene dosage and mRNA stability.

Furthermore, our study revealed that *pcnB* deletion significantly impaired biofilm formation capacity and overall bacterial viability (Figures 2A,B). As a critical virulence phenotype for bacterial colonization and environmental stress resistance, the weakened biofilm formation may be attributed to multiple factors (Guerra et al., 2022): first, the stability of quorum-sensing signaling molecules might decrease due to *pcnB* deletion (Sinha et al., 2018); second, bacteria may undergo metabolic energy redistribution; third, outer membrane integrity might be altered. Meanwhile, the reduced FDA hydrolysis activity further indicates widespread defects in enzymatic activity and metabolic states in Δ*pcnB* strains, which is highly consistent with their growth delay phenotype in minimal medium. These findings provide new experimental evidence for comprehensively understanding the regulatory role of *pcnB* in bacterial physiological metabolism. Although this study establishes the critical role of *pcnB* in virulence plasmid stability, we acknowledge a limitation: The exact mechanism involving the half-life of *aadA* mRNA remains to be elucidated in future work.

## Data Availability

The original contributions presented in the study are included in the article/Supplementary material, further inquiries can be directed to the corresponding authors.
