# Supplementary material for: The poly (A) polymerase pcnB modulates virulence and resistance in Klebsiella pneumoniae by differentially regulating chromosomal mRNA stability and plasmid copy number
Source: Front Microbiol. 2025 Dec 11;16:1709161. doi: 10.3389/fmicb.2025.1709161 (PMC12740245; doi:10.3389/fmicb.2025.1709161)
Supplement: Supplementary file 1 [file Data_Sheet_1.docx]

**Supplementary material**

**Table S1 Plasmids and primers used in this study**

| Plasmids/ primers | Purpose | Sequence (5’ – 3’) | Reference |
| --- | --- | --- | --- |
| pSGKP-km | As a vector with homologous arms and sgRNA that provides target-specific sequenc((Kan^r^) | \ | \ |
| pEcgRNA | Carrying the spectinomycin resistance gene aadA  (Spc^r^) | \ | \ |
| pCasKP-apr | Thermosensitive plasmid expresses Cas9 and lambda Red proteins in K. pneumoniae (Apr^r^) | \ | ^1^ |
| Primers for upstream of *pcnB* | Amplifying the upstream of *pcnB* | Fw:CATTTATGCCTTCATCGGCGAACAG  Rv:CTCTGGTCTATATCGCTCTTGGC | this study |
| Primers for downstream of *pcnB* | Amplifying the downstream of pcnB | Fw:GGTGCACCTCGTAGTGTCAGAC  Rv:CGTGGGCGGGATCTACGACGG | this study |
| Primers for upstream homology arm of *pcnB* | overlap extension PCR | CTGACACTACGAGGTGCACCCTCTGGTCTATATC | this study |
| Primers for downstream homology arm of *pcnB* | overlap extension PCR | AAGAGCGATATAGACCAGAGGGTGCACCTCGTAGTG | this study |
| Primers for reverse sequence of pSGKP-km | homologous recombination | Fw:GCAAACCGCCTCTCCCCGC  Rv:GTATTGGGCGCTCTTCCGC | this study |
| Primers for upstream and downstream homology arms of *pcnB* | homologous recombination | Fw:GCGGGGAGAGGCGGTTTGCCATTTATGCCTTCATCGGCGAACAG  Rv:GCGGAAGAGCGCCCAATACCGTGGGCGGGATCTACGACGG | this study |
| target sequence of *pcnB* | Guiding Cas9 to cleave *pcnB* | TAGTCAATGATTTCCGGCCCGAAC  AAACGTTCGGGCCGGAAATCATTG | this study |
| qPCR primers | Measuring gene expression | *entD*-Fw:AAGATCTGTTCTGGCTGCCC  *entD*-Rv:CTGCCTGAATCCATTGCAGC  *fepD*-Fw:TGTCAAAGCGCTGATTGCAC  *fepD*-Rv:GTGATGACGATCAGGCCGAT  *iroE*-Fw:CAGGTTAGCTTCCGTCTGGG  *iroE*-Rv:AGCAACAAAACAGGGGGACA  *fimA*-Fw:TCTCATCGGTTGGGCGAAAA  *fimA*-Rv:ATGATGTTGGGGCCGTCATT  *fimH*-Fw:CTGTATCTGACGCCGGTGAG  *fimH*-Rv:GGTGATGCCGATGATCGACT  *mrkA*-Fw:ACGTAGTAGGTGAAACGCGC  *mrkA*-Rv:TCTCTCTGCAGCAATGGCAA  *rfbA*-Fw:AAGTAATGTGATGATGGA  *rfbA*-Rv:AATAGCAATGAGAGGTATA  *relA*-Fw:ACAAAAAGGGTATCGTCCGTA  *relA*-Rv:AATCACGCTTGGTATTGCTAATTG  *wbbW*-Fw:TCTATACCAGCCACCATA  *wbbW*-Rv:ATATTATCGCCACTGTCAT  *gapA*-Fw:ATACCGGTCAGTTTGCCGTT  *gapA*-Rv:ACTCCACTCACGGTCGTTTC  *addA*-Fw:TTGGAAACTTCGGCTTCCCC  *addA*-Rv:TCTTCCAACTGATCTGCGCG | this study |
| qPCR primers for 16sRNA | Internal reference | Fw:TTCGATGCAACGCGAAGAAC  Rv:TTTCACAACACGAGCTGACG | this study |

**Table S2 Composition of minimal medium for kp cultivation**

| Composition | Weight (L^-1^) |
| --- | --- |
| KH_2_PO_4_ | 3.0 g |
| NH_4_SO_4_ | 5 g |
| NH_4_Cl | 1 g |
| MgSO_4_·7H_2_O | 0.3 g |
| Na_2_HPO_4_ | 6.8 g |
| NaCl | 0.5 g |
| C₆H₅Na₃O₇ | 1.5 g |
| D-C_6_H_12_O_6_ | 4 g |
| CaCl_2_ | 0.01 g |
| glycerinum | 5 mL |


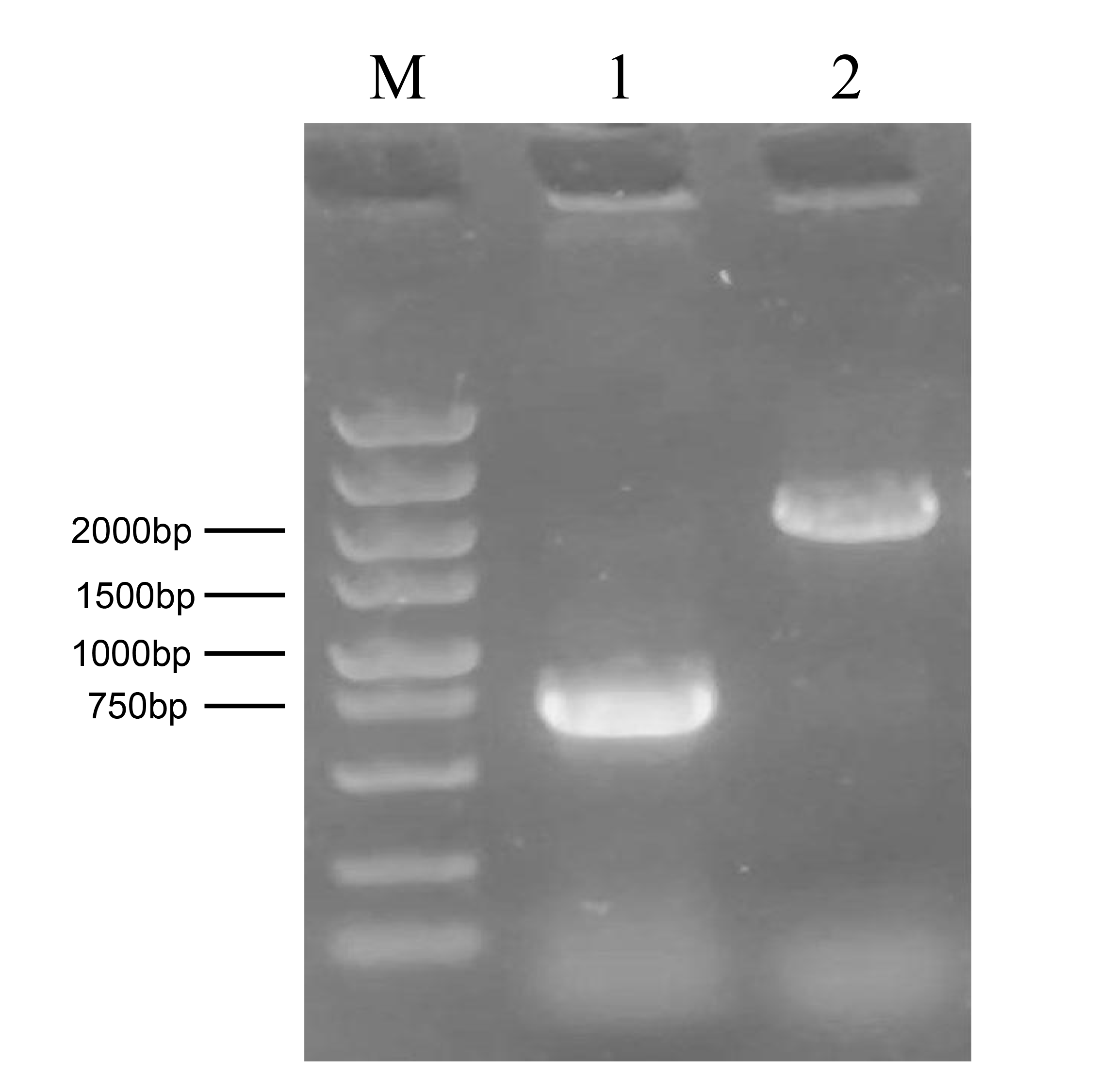


**Figure S1.Validation of pcnB knockout in kp。M:DL5000 DNA Marker;1:ΔpcnB;2:WT**


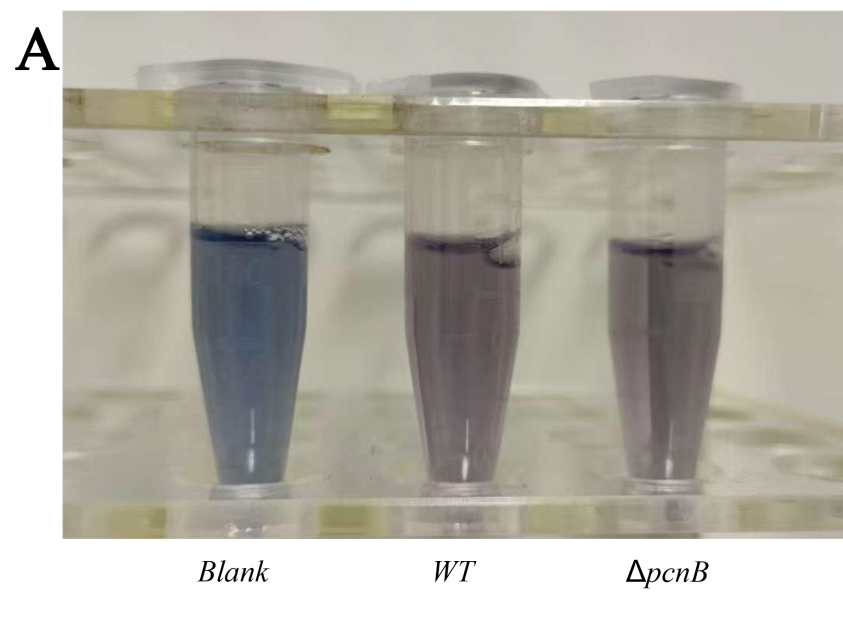

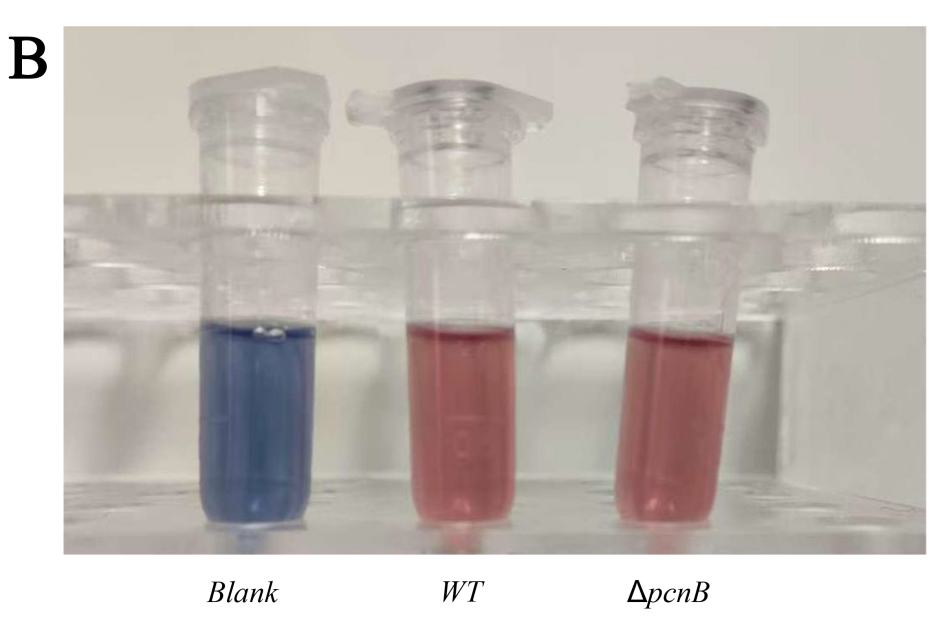


**Figure S2. Qualitative detection of siderophores by the Chrome Azurol S (CAS) assay。A：Exponential Phase；B：Stationary Phase。**

(1) Sun, Q.; Wang, Y.; Dong, N.; Shen, L.; Zhou, H.; Hu, Y.; Gu, D.; Chen, S.; Zhang, R.; Ji, Q. Application of CRISPR/Cas9-Based Genome Editing in Studying the Mechanism of Pandrug Resistance in Klebsiella pneumoniae. *Antimicrobial Agents and Chemotherapy* **2019**.
